# Assessing the effectiveness of a DASH diet in hypertensive patients attending the Ngaoundere Regional Hospital – Cameroon: a case–control study

**DOI:** 10.1017/jns.2023.67

**Published:** 2023-07-25

**Authors:** Mireille Flore D. Kenmoue, Wilfred D. Ngaha, Edith N. Fombang, Majeste M. Pahane, Stephane Simeu

**Affiliations:** 1 Department of Food Sciences and Nutrition, National School of Agro-Industrial Sciences, The University of Ngaoundere, P.O. BOX 455, Ngaoundere, Cameroon; 2 Department of Processing and Quality Control, Institute of Fisheries and Aquatic Sciences, The University of Douala, P.O. BOX 7236, Douala-Bassa, Douala, Cameroon; 3 Service of Cardiology, Regional Hospital of Ngaoundere, P.O. BOX 29 Meiganga, Ngaoundere, Cameroon

**Keywords:** Blood lipid profile, Body mass index, Dash diet, Hypertension, Ngaoundere Regional Hospital, BMI, body mass index, BP, blood pressure, DASH, Dietary Approaches to Stop Hypertension, DBP, diastolic blood pressure, DTEI, daily total energy intake, HDL-c, high-density lipoprotein cholesterol, LDL-c, low-density lipoprotein cholesterol, NRH, Ngaoundere Regional Hospital, SBP, systolic blood pressure

## Abstract

Hypertension remains a public health issue in Cameroon, though lifestyle and dietetic measures are the main approaches for the prevention and management of hypertension. The present study aimed at evaluating the impact of a Dietary Approaches to Stop Hypertension (DASH) diet using local foodstuffs on the status of hypertensive patients at the Ngaoundere Regional Hospital. A case–control study was carried out with 160 hypertensive patients divided into two groups, a test and a control group. A food questionnaire was used to evaluate the food habits of patients and design the sheet of the DASH diet to provide a maximum of 2000 kcal/d. The DASH diet was administered to the test group (eighty-eight patients), while the control group (seventy-two patients) consumed their normal diet. Both groups were followed up for 8 weeks. The systolic and diastolic blood pressures (SBP, DBP), body mass index (BMI), triglycerides, HDL**-**c, LDL-c and total-cholesterol levels of patients of the two groups were measured before and after the intervention. The results indicate that the DASH diet improves all the markers of hypertension in the test group with significant decreases in BMI, SBP, DBP, LDL-c and total-cholesterol. Patients of the control group had fourteen and seven times more risk of having increased systolic and diastolic pressures, respectively, and are thus exposed to hypertension complications. The DASH diet established in this study is therefore effective for the management of hypertension.

## Introduction

Hypertension is a pathologic state characterised by permanent high blood pressure on the walls of vessels, higher than 140 mmHg for systolic blood pressure (SBP) and/or higher than 90 mmHg for diastolic blood pressure (DBP)^(1)^. This high pressure can damage the walls of the blood vessels in the long term. The risk factors of hypertension are mainly associated with food practices and lifestyle, particularly diets rich in salt and fats, alcoholism, tobacco and low or absence of physical exercises, low consumption of fruit and vegetables, aging, family history and overweight^(1)^.

Hypertension affects about a billion people around the world and causes more than 10 million deaths each year, with a prevalence that does not significantly vary between urban (32⋅6 %) and rural (34⋅3 %) areas^(1)^. Women are more affected (37 %) than men (30⋅2 %), and the prevalence increases with age, affecting 19⋅2 % population from 20 to 34 years old, 53⋅8 % of 40–60 years and 72⋅2 % of the population group from 65 years^(2)^. Hypertensive patients are exposed to associated pathologies, notably renal and cardiac failures, arterial aneurism, aortic dissection, arrhythmia and cognitive and mental disorder^(1)^. However, a slight reduction in blood pressure could considerably decrease morbidity and mortality related to these related diseases and complications, for example, a decrease of 2 mmHg in the DBP have been reported to reduce by 17 % the prevalence of hypertension, by 6 % the risks of coronary diseases and by 15 % the risks of cerebrovascular accidents^(3)^.

The management of hypertension by medical prescription of antihypertensive treatment and/or by lifestyle-dietetic measures (weight loss, reduction of salt intake, regular physical activity, etc.) aimed at decreasing blood pressure and other risk factors of hypertension like overweight and hyperlipidaemia^(4)^. Accordingly, the Dietary Approaches to Stop Hypertension (DASH) diet developed in the 1990s in the United States to fight against hypertension, recommends a diet rich in fruits and vegetables, whole cereals, skimmed dairy products, legumes/grains and low in fat, sodium (Na^+^) and refined sugars, accompanied with regular physical activity^(5)^. Several studies have shown the effectiveness of this diet in the management of hypertension, with a significant decrease of the SBP and DBP, weight, waist circumference and serum level of total- and LDL-cholesterol^(6–11)^, cardiovascular and renal diseases, metabolic syndrome, gestational diabetes and colorectal cancer^(12)^.

In Cameroun, more than 25 % of the general population are hypertensive^(13)^, and the hypertension management is challenging because of the lack of financial resources, customs, beliefs and the negative perception of the disease^(14)^ as well as lack of information/education on the hypertension prevention and management. Some of these constraints can however be circumvented by a DASH diet based on available local resources and taking in account the food habits and practices of the patients. The general objective of the present study was to evaluate the effectiveness of a DASH diet with local foodstuffs in nutritional management of hypertension among patients attending the Ngaoundere Regional Hospital (NRH).

## Materials and methods

### Sampling

The intervention was a case–control prospective experimental study carried out for eight weeks from December 2021 to February 2022 on hypertensive patients attending the cardiology unit of the NRH. The minimal sample size (*N*) was calculated using the formula of Lorenz (Eq. 1):1
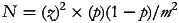
where *z* is the factor to reach 95 % of interval of confidence (1⋅96); *p* is the prevalence of hypertension (0⋅25) and *m* is the margin of error (0⋅05).

The sample size obtained after calculation was 288 subjects. But due to the limited time for the study, a systematic sampling of all the patients having given their consent led to the recruitment of 160 patients, a limitation for this study according to the power sample size. The patients recruited were of both genders and were divided into two groups, a test group of eighty-eight patients following the DASH diet, and a control group of seventy-two patients taking their normal diet with nutritional advices.

### Nutritional intervention

Patients were received individually in a consultation room where they could express themselves freely and in all confidence. After obtaining the informed consent, they were subjected to an interview using a questionnaire aimed to collect data on food practices, lifestyle, history of hypertension, but also socio-demographic characteristics like age, gender, marital status, level of education and profession. The data on the food practices was precisely related to food regularly consumed, daily food frequency, food cooking mode, place of consumption of meals (family, restaurant, fast food), and daily frequency and quantity of water consumed. Concerning lifestyle, information on alcohol consumption, smoking tobacco and the practice of physical activity (type and frequency) were investigated.

The patients then received nutritional advice, with those of the test group additionally receiving a 7-d diet plan, established based on the season's foodstuffs available, and partitioned into breakfast (7–9 a.m.), lunch (12–2 p.m.) and dinner (5–7 p.m.). An appointment for follow-up was set at 2 weeks’ interval, but also aiming to evaluate the diet prescribed and make some adjustments where necessary.

### Nutritional management

The intervention consisted in administering to the test group, for 8 weeks, a DASH diet (Appendix 1) built up using the results of survey on their food habits and to be followed-up at home, coupled with the practice of a moderate physical activities (walking, bicycle or pastoral work) at least 30 min per day at least three times per week. This DASH diet was rich in fruits, vegetables, skimmed or partially skimmed dairy products, dietary fibres, potassium, calcium and magnesium, moderate in fish, lean meat and poultry, leguminous plants, seeds and dry fruits with hulls, and low in total fat, saturated fatty acids and cholesterol.

The daily total energy intake (DTEI) was estimated based on recommendations of a DASH diet, 2000 kcal divided in three meals and two snacks between the meals. The portions of meals were estimated using food composition tables. Lipid intake accounted for 27 % of the DTEI, cooking oil recommended was soybean oil. Carbohydrate and protein intake were respectively 55 and 18 % of the DTEI. It was recommended to the patients to consume about 2⋅3 g (a teaspoon) of salt (sodium chloride) per day, avoiding monosodium glutamate in their meals, to use not more than six tablespoons of oil in the meals, which should be added at the end of cooking, avoid adding salt to already cooked dishes and to use flavour alternatives like aromatic herbs and spices, and also to consume 2⋅5 to 3 l of water per day. Alcohol intake was limited to a half glass of red wine per day.

Concerning the qualitative aspect, this diet considered the geographic context by preferring foodstuffs resulting from local agriculture. The consumption of fruits and vegetables locally available during the season was strongly recommended (moringa leaves (*Moringa oleifera*), *zom/njapche/koumbi* leaves (*Solanum nigrum*), *kenen kenen* leaves (*Corchorus olitorus*), water melon, orange, apple, banana, tangerine, carrot, French beans, cucumbers, okra, etc.). Since the dietary intakes of the patients of the control group were not followed-up and assessed, their energy intake was not estimated.

### Nutritional assessment

The assessment of the impact of nutritional intervention was done by measuring SBP and DBP, calculating the body mass index (BMI) (kg/m^2^) (Eq. 2), and determining the lipid profile (triglycerides, total-cholesterol, LDL-c and HDL-c) of patients at the beginning and at the end of the 8 weeks of experimentation. BMI used to evaluate the risk of health-related problems associated with overweight and obesity was obtained from the weight (kg) of the patient measured using a balance with impedance-meter (Kenlee) of a capacity of 160 kg with a precision of 0⋅1 kg, and height (m) using a UNICEF removable height gauge of a range of 2⋅20 m with a precision of 0⋅01 m.2
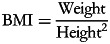


Nutritional status corresponding to BMI values (kg/m^2^) was ranged as follows: <18⋅5: Underweight; 18⋅5–24⋅9: Normal; 25–29⋅9: Overweight; 30–34⋅9: Primary obesity; 35–39⋅9: Secondary obesity; ≥40: Tertiary Obesity (morbid).

SBP and DBP (mmHg) were measured using an electronic blood pressure monitor (OMRON M6 Confort [HEM-7321-E]). The measurements were done three times with an interval of 1 min between two measurements on sitting patients, at rest and not stressed, the armband of the monitor being placed at the level of the non-dominant arm (left arm for the right-handed and right arm for the left-handed persons). Concerning lipid parameters, blood sample was collected by a nurse, labelled and conveyed to the laboratory of Biomedical Analysis of the NRH for the analysis of serum triglycerides, total, HDL-c and LDL-c levels.

### Statistical analysis

The survey sheet was designed using the software Sphinx Plus² – Edition Lexica-V5, and the data analysed using SPSS-v20 software. The qualitative variables were expressed in absolute and relative frequency while the quantitative variables were expressed as mean ± standard deviation for the data with a normal distribution, or as medians with the interquartile intervals for the data not having a normal distribution. The impact of the intervention was evaluated by the test Chi-square test of Mann–Whitney on two series of paired qualitative data. The threshold of signification was fixed at *α* = 0⋅05.

### Ethical considerations

This study was conducted according to the guidelines laid down in the Declaration of Helsinki and all procedures involving human subjects were approved by the Direction of Regional Hospital of Ngaoundere by delivering an authorisation of research. Appropriate protocols for protecting the rights and privacy of all the participants were applied during the nutritional intervention. Verbal informed consent was obtained from all subjects. Verbal consent was controlled and formally recorded. The agreement clearly stated that participation is not compulsory, participants are free to withdraw from the study at any time or step, and full disclosure of study requirements and risks of the study. The anonymity of the participants was guaranteed, as well as the confidentiality of data collected. In addition, this study did not harm the physical integrity of the patients and did not interfere with their clinical follow-up.

## Results and discussion

### Socio-demographic characteristics of the study population

The socio-demographic characteristics of the patients are presented in Table 1, with a predominance of men (51⋅2 %) compared to women (48⋅8 %), and this can be due to the fact that android obesity localised in the upper part (abdomen) of the body is most frequent in men is a risk factor of hypertension^(2)^. A study carried out in the town of Ngaoundere in 2020 also revealed a high predominance of men (63⋅8 %) among the hypertensive patients^(15)^.

**Table 1.** Socio-demographic characteristic of studied patients

Variable	Modality	Number	Frequency (%)
Age range (year)	20–39	12	7⋅5
	40–59	90	56⋅3
	≥60	58	36⋅2
Gender	Male	82	51⋅2
	Female	78	48⋅8
Marital status	Married	113	70⋅6
	Single	10	13⋅0
	Divorcee	6	3⋅8
	Widower	42	12⋅6
Level of education	None	52	32⋅0
	Primary	50	31⋅3
	Secondary	34	21⋅2
	High	24	15⋅0
Occupation	unemployed	64	40⋅0
	Public servant	34	21⋅3
	Pensioner	18	11⋅3
	Contractor	6	3⋅7
	Merchant	20	12⋅5
	Farmer	18	11⋅2

The patients were mainly married (70⋅6 %) (Table 1), which is in line with the advanced age of many of them, as 92⋅5 % of the participants were 40 years old and more. Even though an advanced age is a risk factor of hypertension related to the ageing of the blood vessels, results show that patients of 40–59 years (56⋅3 %) represented the higher proportion of the studied population than those from 60 years and above (36⋅2 %). Thus, this can be justified by another risk factor of hypertension associated with age, poor stress management^(16)^. Indeed, contrary to patients 60 years old who are pensioners for the majority, those aged 40–59 years are still active, and thus undergo the pressure and the stress related to the long working days. A study carried out in the Garoua town in Cameroon established that the age group of 46–65 years were the majority in population of hypertensive patients^(17)^. From Table 1, 40 % of patients were unemployed and 11⋅3 % were pensioners, 48⋅7 % of the participants had a job, justifying once more that the stress related to the working days could be a risk factor of hypertension. For the pensioners’ patients, hypertension could be associated with their age with shrinkage and stiffness of their blood vessels.

Concerning the level of education, 32 % of the patients had never gone to formal school or follow certifying education and 313 % have primary school level. Level of education is a significant factor in the development of nutritional intervention programs. Because of their low level of education, about two-third of participants can neither speak, read nor write English and/or French, the two official languages in Cameroon. They are consequently unable to understand the messages and sensitising in the media on hypertension. Moreover, they do not measure the importance of nutrition in their lifestyle. In fact, it was shown that people who received a minimum of education are generally more advised than those who did not, and lack of education can negatively affect the decisions about food choices^(18)^. In fact, the higher the level education, the lower is the prevalence of malnutrition among women and their families^(19)^.

### Physical activity and hypertension antecedents of the participants

The type, frequency and duration of physical activity by the participants, as well as the history of their hypertension are presented in Table 2. Most of the patients (67⋅5 %) did not practice any physical activity before the study, and only 15 % practised more than three times per week, which exposes them to the disease as physical inactivity is one of the risk factors of hypertension^(20,21)^. Moreover, 61⋅3 % of patients had family history of hypertension (Table 2), one of the causes of occurrence of hypertension^(1)^. About half of the patients (41⋅9 %) were diagnosed less than one year back, while 34⋅4 % of patients were diagnosed more than 5 years back showing that hypertension is diagnosed late in the town of Ngaoundere, which can be related to the lack of information of the patients on the symptoms of the disease, their low level of education, but especially the low rate of hospitals visits by the population.

**Table 2.** Physical activity, family history and period of diagnosis of hypertension

Variable	Modality	Number	Frequency (%)
Physical activity	None	108	67⋅5
	Walking	38	23⋅8
	Fitness	8	5⋅0
	Pastoral work	6	3⋅7
Weekly physical activity frequency	<3 times	28	17⋅5
	>3 times	24	15⋅0
Duration of physical activity (min)	<30	16	10⋅0
	>30	36	22⋅5
Family history of hypertension	Yes	98	61⋅3
	No	38	23⋅7
	No idea	24	15⋅0
Period of diagnosis of hypertension (year)	≤1	67	41⋅9
	1–5	38	23⋅7
	5–10	18	11⋅3
	>10	37	23⋅1

### Lifestyle-dietary practices of surveyed patients

Results on food practices and lifestyle of the patients (Table 3) indicate more than one-third of the patient consume salt in higher quantities for their pathologic state, with only 5 % of the patients consuming three fruits and vegetables per day and 5 % using olive oil to prepare their meals. A total of 85 % of patients use palm oil, refined or not, because of its availability and its accessibility in the markets, though it contains significant proportions of saturated fatty acids^(22)^. Indeed, it is recommended to hypertensive patients to significantly reduce their intake of table salt, as there is a high correlation between a diet rich in Na^+^ and the occurrence of hypertension^(23)^. Moreover, with their high content in vitamins, minerals, antioxidants and others bioactive compounds, fruits and vegetables are strongly recommended in the management of hypertension, and it is the same for olive oil with its wealth in polyunsaturated fatty acids^(24)^.

**Table 3.** Lifestyle-dietary practices of the participants

Variable	Modality	Number	Frequency (%)
Salt consumption	Normal	50	31⋅3
	Low	106	66⋅2
	No	4	2⋅5
Number of cooked or raw vegetable/day	None	54	33⋅8
	1	56	35⋅0
	2	42	26⋅2
	3	8	5⋅0
Cooking oil	Refined palm	114	71⋅3
	Red palm	22	13⋅7
	Cotton	16	10⋅0
	Olive	8	5⋅0
Whole cereals	Yes	68	42⋅5
	No	76	47⋅5
Fish consumption/week	Never	6	3⋅8
	1 time	40	25⋅0
	2 times	38	23⋅7
	3 times	32	20⋅0
	>3 times	20	12⋅5
Red meat consumption/week	Never	6	3⋅8
	1 time	24	15⋅0
	2 times	16	10⋅0
	3 times	52	32⋅5
	>3 times	62	38⋅7
Consumption of alcohol	Yes	22	13⋅7
	No	113	70⋅6
	Sometimes	25	15⋅6
Smoking tobacco	Yes	8	5⋅0
	No	150	93⋅7
	Sometimes	2	1⋅3

Cattle breeding is the major activity in the study area, then beef meat is available in the local markets, which explains why 71⋅2 % of the patients consume meat at least three times per week, contrary to fish which is a little bit scarce, and thus less consumed with only 32⋅5 % of participants who eat fish at least three times per week. This result suggests that the feeding habits of the patients are likely to expose them to complications, because red meats must be reduced in the diets for chronic diseases in general and DASH diet. Moreover, fish, in addition, is also a source of unsaturated fatty acids which have properties proven in the management of hypertension^(25)^.

Among the patients, 42⋅5 % consumed whole cereals. Cereals are famous for their high content in dietary fibres which are implicated through several mechanisms in the reduction of blood cholesterol and sugar in the body.

Concerning the lifestyle, 70⋅6 % of patients did not consume alcohol and 93⋅7 % of patients did not consume tobacco. However, 13⋅7 % of the patients frequently consumed alcohol and 5 % consumed tobacco. Even though smoking and consumption of alcohol are high risk factors of hypertension^(26,27)^, they present a low risk for the patients attending NRH nutrition unit.

### Impact of the DASH diet on nutritional status of patients

BMI of patients at the beginning and at the end of the intervention were calculated and the results compiled in Table 4 indicate that the DASH diet has improved the nutritional status of patients of test group by significantly reducing their BMI. Indeed, the percentage of patients suffering from obesity dropped from 47⋅7 % before intervention to 22⋅7 % after intervention, corresponding to a global drop of about 43 % of obese patients. Furthermore, there was an increase in the proportion of the patients having a normal BMI from 18⋅2 to 36⋅4 %, corresponding to a global increase of 50 % of patients of the normal weight. This can be explained by the limited amount of energy supplied (2000 kcal/d) by the dash diet used. This DASH diet is also rich in fruit and vegetables containing antioxidants that may influence fat metabolism^(28)^, and also rich in whole cereals containing dietary fibres implicated in the reduction of blood glucose and lipid levels, thus limiting their storage in the form of energy^(29)^. This diet combined with the practice of physical activity, largely contributed to the reduction of body weight of the patients, as reported in a similar study carried out in Algeria^(23)^.

**Table 4.** Effect of the diet on the body mass index of patients

		Body mass index (kg/m^2^)/Frequency of patients (%)					
Groups		18⋅5–24⋅9	25–29⋅9	30–34⋅9	35–39⋅9	>40	
Case	Before	18⋅2	34⋅1	31⋅8	13⋅6	2⋅3	***P* = 0⋅004**
	After	36⋅4	40⋅9	18⋅2	4⋅5	0	
Control	Before	38⋅9	33⋅3	16⋅7	5⋅6	5⋅6	*P* = 0⋅997
	After	36⋅1	36⋅1	16⋅7	5⋅6	5⋅6	

Test group *n* 88; Control group *n* 72.

Bold values indicate that the difference is statistically significant (*P*-value < 0.05).

### Impact of DASH diet of blood pressure of the patients

**Fig. 1.**
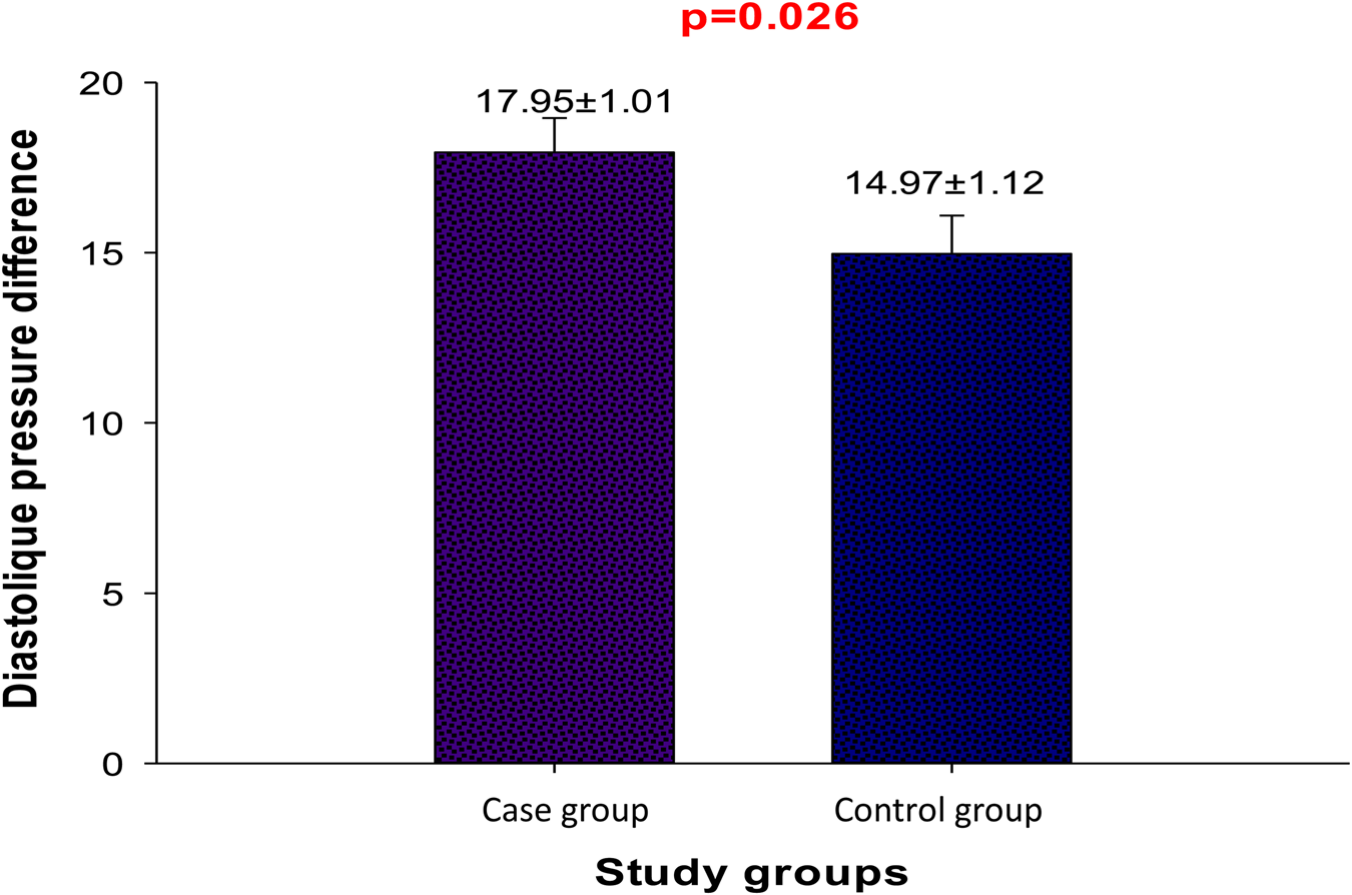
Comparison of diastolic blood pressure variations between case and control groups.

SBP and DBP of the patients before and after the intervention are presented in Table 5. There is a significant decrease (*P* < 0⋅05) in the SBP and DBP in both test and control groups. This reduction is of about 28 and 18 mmHg, respectively, for SBP and DBP in the test group, 27 and 15 mmHg in the control group and can be explained by the fact that all the patients in the test and control groups, were under antihypertensive treatment during the period of the study, which would have contributed to this significant decrease in blood pressure. However, a comparison of the average values of the variations of DBP (Fig. 1) shows a higher decrease in the test (*P* < 0⋅05) compare to the control group. This could be justified by the benefits of the experimental diet, in particular the reduction of Na^+^ intake and the consumption of fruits, vegetables and fish^(23)^. The regular consumption of fish which is rich in ω-3 polyunsaturated fatty acids (3 times per week) and vegetable oils rich in unsaturated fatty acids leads to the decrease in the blood pressure, and can be recommended among hypertensive patients^(25)^.

**Table 5.** Systolic and diastolic blood pressures before and after the diet

Variable		Before	After	Difference	*P*-value
SBP (mmHg)	Case	154⋅0 ± 1⋅6	126⋅1 ± 1⋅1	−27⋅9 ± 1⋅4	***P* < 0⋅001**
	Control	156⋅5 ± 1⋅3	129⋅9 ± 0⋅9	−26⋅5 ± 1⋅2	***P* < 0⋅001**
DBP (mmHg)	Case	100⋅1 ± 1⋅2	82⋅1 ± 0⋅7	−18⋅0 ± 1⋅1	***P* < 0⋅001**
	Control	101⋅7 ± 1⋅5	86⋅6 ± 0⋅9	−15⋅0 ± 1⋅7	***P* < 0⋅001**

SBP, systolic blood pressure; DBP, diastolic blood pressure.

Bold values indicate that the difference is statistically significant (*P*-value < 0.05).

### DASH diet and severity of systolic and diastolic blood pressures

The impact of the DASH diet on the evolution of the severity of hypertension is presented in Table 6. For the SBP, there is a significant improvement (*P* < 0⋅05) in the test group, with the prevalence of the patients at the advanced stages (grades 1, 2 and 3) passing from 88⋅7 % at the beginning of the study to 4⋅5 %, with no patient at the grades 2 and 3 at the end of the study. The same trend was observed for the DBP with the proportion of patients with advanced stage of the disease passing from 84 to 9 %, without patient in grade 3 at the end of the study. These results confirm once more the effectiveness of this DASH diet in the management of hypertension.

**Table 6.** Effect of diet on the severity of systolic and diastolic blood pressures

Systolic blood pressure (mmHg)/Frequency of patients (%)								
Groups		Optimal < 120	Normal <130	Normal h^a^ 130–139	Grade 1 140–159	Grade 2 160–179	Grade 3 >180	*P*-value
Case	Before	0	2⋅3	9⋅1	61⋅4	25	2⋅3	***P* < 0⋅001**
	After	22⋅7	38⋅6	34⋅1	4⋅5	0	0	
Control	Before	0	0	8⋅3	58⋅3	16⋅7	16⋅7	***P* < 0⋅001**
	After	11⋅1	5⋅6	50	33⋅3	0	0	
Diastolic Blood Pressure (mmHg)/Frequency of patients (%)								
Groups		Optimal <80	Normal <85	Normal h^a^ 86–89	Grade 1 90–99	Grade 2 99–109	Grade 3 >110	*P*-value
Case	Before	2⋅3	2⋅3	11⋅4	29⋅5	31⋅8	22⋅7	***P* < 0⋅001**
	After	22⋅7	36⋅4	31⋅8	4⋅5	4⋅5	0	
Control	Before	2⋅8	0	5⋅6	44⋅4	25	22⋅2	***P* < 0⋅001**
	After	5⋅6	11⋅1	50	33⋅3	0	0	

a Normal high/Test group *n* 88; Control group *n* 72.

Bold values indicate that the difference is statistically significant (*P*-value < 0.05).

The decrease in blood pressure observed in this study may also be due to the richness of the elaborated DASH diet in calcium (Ca^2+^), magnesium (Mg^2+^) and potassium (K^+^) which are vasodilator minerals. Indeed, the movement of Ca^2+^ on both sides of the cellular membrane is accompanied by a reduction of the blood pressure and a decrease in the intracellular concentration of Ca^2+^, causing a relaxation of vascular fibres and thus a vasodilatation^(30)^. Mg^2+^, on the other hand, acts on vascular tonicity through mediators like nitric oxide (NO), endotheline-1 and the derivatives of prostaglandins released by the endothelium and lowers the blood pressure^(31)^. For the case of K^+^, it reduces the blood pressure by acting on the urinary elimination of Na^+^ and water^(32)^. K^+^ would have also the capacity to recover noradrenalin at the level of nervous terminations and consequently is effective as a vasodilator leading to a reduction of the blood pressure^(33)^.

### Effect of DASH diet on the lipid profile of the patients

**Fig. 2.**
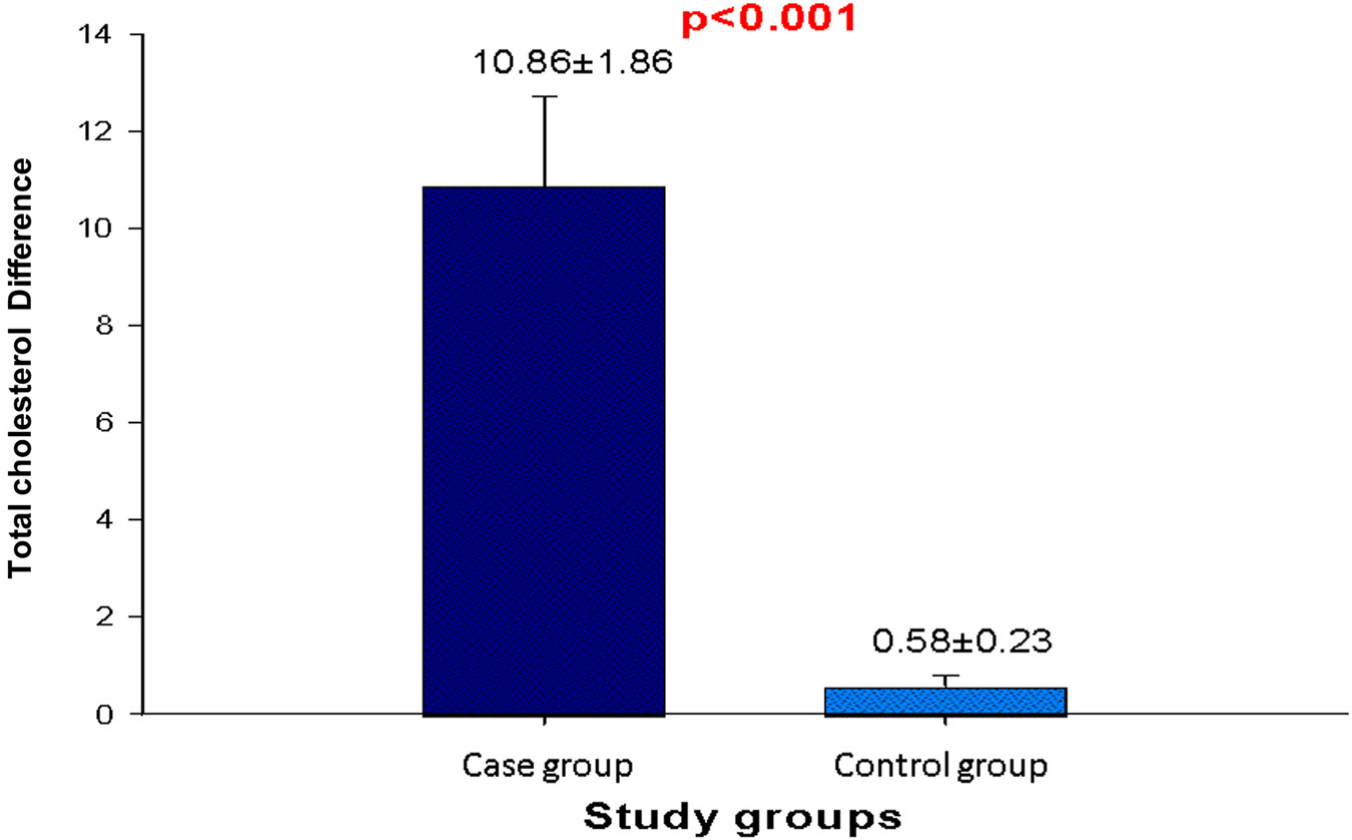
Comparison of variation of total-cholesterol level between case and control groups.

Lipid profile of the patients before and after the study is presented in Table 7. Except triglyceride concentration, DASH diet has a significant impact (*P* < 0⋅05) on the lipid parameters. Total-cholesterol and LDL-c levels decrease for the patients of the test group based on average by 10⋅86 and 9⋅56 mg/dl, respectively, while their HDL-c level increased by 4⋅34 mg/dl. Total-cholesterol level significantly (*P* < 0⋅05) decreases in the test group than the control group (Fig. 2). These results highlight the effectiveness of the established DASH diet using local foodstuffs from Ngaoundere in the management of hypertension and its risk factors. Indeed, this drop in total- and LDL-cholesterol accompanied by an increase in the rate of HDL-cholesterol is the result of a diet rich in dietary fibres, antioxidants and unsaturated fatty acids from foodstuffs selected.

**Table 7.** Lipid profile of patients before and after the intervention

Variable (mg/dl)	Groups	Before	After	Difference	*P*-value	Norm
Total-cholesterol	Case	160⋅8 ± 5⋅1	149⋅9 ± 3⋅9	−10⋅9 ± 1⋅9	***P* < 0⋅001**	<250
	Control	164⋅7 ± 4⋅1	164⋅1 ± 3⋅9	−0⋅6 ± 0⋅2	***P* = 0⋅02**	
LDL-cholesterol	Case	114⋅0 ± 2⋅4	104⋅4 ± 1⋅7	−9⋅6 ± 1⋅3	***P* < 0⋅001**	<130
	Control	101⋅2 ± 2⋅9	100⋅1 ± 2⋅8	−0⋅1 ± 0⋅0	*P* = 0⋅251	
HDL-cholesterol	Case	69⋅9 ± 12⋅9	74⋅20 ± 12⋅9	+4⋅3 ± 0⋅5	***P* < 0⋅001**	35–80
	Control	50⋅0 ± 1⋅3	50⋅1 ± 1⋅3	+0⋅1 ± 0⋅0	*P* = 0⋅073	
Triglycerides	Case	103⋅7 ± 3⋅3	102⋅0 ± 2⋅9	−1⋅7 ± 0⋅5	*P* = 0⋅14	35–160
	Control	141⋅2 ± 6⋅5	141⋅3 ± 6⋅5	−0⋅1 ± 0⋅0	*P* = 0⋅461	

Bold values indicate that the difference is statistically significant (*P*-value < 0.05).

### Risk of exposure of patients

Level of risk of exposure to hypertension (increasing in SBP and DBP) according to the observance of DASH diet among patients is presented in Table 8. Compared to the patients of the test group who received DASH diet, the patients of the control group had fourteen times more risk of increasing the SBP and seven times more risk for the DBP, which also increases the risk of complications. In fact, the DASH diet is high in antioxidants contained in fruits, vegetables and whole grains, and this diet raised antioxidant capacity compared with that of usual diets. The DASH diet may lower BP by raising antioxidant capacity and improving the balance between antioxidant defences and oxidative stress. A study has besides reported that antioxidant capacity, measured by the FRAP assay, was higher in lean normotensives than in obese hypertensives on their usual diets^(34)^.

**Table 8.** Relative risk of hypertension in the participants

Systolic blood pressure (mmHg)					
		Normal (<135)	Abnormal (>135)	*P*-value	Relative risk
Groups	Case	76 (77⋅6)	12 (19⋅4)	*P* < 0⋅001	14⋅4
	Control	22 (22⋅4)	50 (80⋅6)		
Diastolic blood pressure (mmHg)					
		Normal (<85)	Abnormal (>85)	*P*-value	Relative risk
Groups	Case	62 (77⋅5)	26 (32⋅5)	*P* < 0⋅001	7⋅2
	Control	18 (22⋅5)	54 (67⋅5)		

## Conclusion

The good application of a DASH diet established from available food resources in the town of Ngaoundere contributes effectively to the management of hypertension, by improving the physiological (SBP and DBP), anthropometric (BMI) and lipid (total-, HDL- and LDL-cholesterol) parameters of the hypertensive patients. The application of DASH diet proposed reduces the risk of complications mainly by fourteen times the risk links to an increasing of the SBP and by seven times the risk links to an increasing of the DBP.

## Appendix 1: Seven-day diet plan for the test group

**Table:**

Meals/days	Breakfast (7–9 a.m.)	Lunch (12–2 p.m.)	Dinner (5–7 p.m.)
Day 1	Uncooked salad (½ carrot, lettuce, tomato, onion, cucumber, ½ avocado + (vinaigrette = lemon and 1 tbsp soyabean oil) + 1 glass of milk + 2 medium ripe bananas	3 slices of yam in broth with steamed carp + 2 medium ripe bananas + 1 medium orange	2 white eggplants, 1 small cucumber, 2 medium ripe bananas, 1 slice of watermelon, 1 glass of milk
Day 2		*Kenen kenen* smoked fish + 10 tbsp corn flour fufu (1 piece of meat) + 1 medium orange + 2 dates	2 white eggplants, 1 small cucumber, 2 medium ripe bananas, 1 slice of watermelon, 1 glass of milk
Day 3	*Kenen kenen* smoked fish + 10 tbsp corn flour fufu (1 piece of meat) + 1 medium orange + 2 dates	Carp broth + 4 small pieces of steamed sweet potato + 1 slice of watermelon	Carp broth + 4 small pieces of steamed sweet potato + 1 slice of watermelon
Day 4	1⋅5 ball of wholemeal bread + 1 glass of milk + 1 slice of watermelon + 1 ripe banana	Meat okra sauce + 10 tbsp corn flour fufu (1 piece of meat) + 1 medium orange	2 white eggplants, 1 small cucumber, 2 medium ripe bananas, 1 slice of watermelon, 1 glass of milk
Day 5	½ avocado with onions, tomato, cucumber, lettuce, + lemon and 1 tbsp soybean oil) + 1 ball of wholemeal bread + glass of milk + 1 slice of semi-ripe papaya + 1 medium ripe banana	Stir-fried moringa with smoked fish and 1 glass of cowpea + 4 small pieces of steamed sweet potato + 1 slice of watermelon	Uncooked salad (half carrot, lettuce, tomato, onion, cucumber, ½ avocado + lemon + 1 tbsp of soyabean oil) + 1 ball of wholemeal bread + 1 glass of milk + 1 slice of semi-ripe papaya
Day 6		Stir-fried beans with mixed vegetables + 3 slices of steamed white yams + 1 slice of watermelon + 2 dates	
Day 7	1½ balls of wholemeal bread + 1 glass of milk + 1 slice of watermelon +1 sweet banana finger	Stir-fried zom with smoked fish + 4 small pieces of steamed sweet potato + 2 ripe bananas + 1 medium orange	Fried zom with smoked fish + 1 small piece of steamed sweet potato + 1 ripe banana + 1 medium orange

tbsp., tablespoon; *Kenen kenen*, *Corchorus olitorus*; *Zom*, *Solanum nigrum.*
